# Adolescence/adult onset MTHFR deficiency may manifest as isolated and treatable distinct neuro-psychiatric syndromes

**DOI:** 10.1186/s13023-018-0767-9

**Published:** 2018-02-01

**Authors:** Ana Gales, Marion Masingue, Stephanie Millecamps, Stephane Giraudier, Laure Grosliere, Claude Adam, Claudio Salim, Vincent Navarro, Yann Nadjar

**Affiliations:** 1 AP-HP, GH Pitié-Salpêtrière-Charles Foix, Epileptology Unit, Paris, France; 2 Neurology Department, Reference Center for Lysosomal Diseases, Neurogenetics and Metabolism Unit, AP-HP, GH Pitié-Salpêtrière-Charles Foix, Paris, France; 3 UPMC University, Institut du Cerveau et de la Moelle épinière, Paris, France; 4 AP-HP, GH Henri Mondor, Laboratoire d’Hématologie, Créteil, France; 5 UPEC University, Creteil, France; 6 INSERM U1131, Saint Louis Hospital, Paris, France; 7 Orphan Europe, Puteaux, France; 8 Sorbonne Universités UMRS1127, INSERM U1127, CNRS UMR7225, Institut du Cerveau et de la Moelle épinière, Paris, France

**Keywords:** Metabolic disease, Clinical neurology examination, All epilepsy/seizures, Gait disorders/ataxia, MRI

## Abstract

5,10-Methylene-tetrahydrofolate reductase (MTHFR) deficiency is a genetic disorder that can occur at any age and can be easily detected by increased homocysteinemia. In adolescence/adult onset forms, the clinical picture is often complex with association of various neurological features and thrombosis.

Here we report the cases of two adult siblings who experienced focal epilepsy at 18 years old as a first disease manifestation, without other symptom during several years. Upon diagnosis, both patients received metabolic treatment comprising B9, B12 and betaine which has stopped the occurrence of seizures, allowing discontinuation of anti-epileptic drugs.

Among 24 reviewed adolescent/adult onset patients with MTHFR deficiency in the literature, clinical manifestations included gait disorder (96%, from motor central or peripheral origin), cognitive decline (74%), epileptic syndromes (50%), encephalopathy (30%), psychotic symptoms (17%), and thrombotic events (21%). A total of 41% presented a single neurological manifestation that could stay isolated during at least 3 years, delaying achievement of the diagnosis. Brain MRI showed a mostly periventricular white matter changes in 71% of cases. All patients stabilized or improved following metabolic treatment.

Despite being rare, adolescence/adult onset MTHFR deficiency can nevertheless be successfully treated. Therefore, homocysteinemia should be tested in various unexplained neuro-psychiatric syndromes like epilepsy or spastic paraparesis, even if isolated, since waiting for completion of the clinical picture is likely to increase the risk of irreversible neurological damage.

**Electronic supplementary material:**

The online version of this article (10.1186/s13023-018-0767-9) contains supplementary material, which is available to authorized users.

## Background

5,10-Methylene-tetrahydrofolate reductase (MTHFR) deficiency (OMIM number #607093) is a rare disorder affecting the metabolism of folate and sulfur-containing amino acids [1]. The enzymatic deficiency results in a reduction in synthesis of 5- methyl-tetrahydrofolate (5MTHF), the biologically active form of folate, which is a cofactor necessary for the re-methylation of homocysteine into methionine. Biological hallmarks are moderately low plasmatic folate levels, hyperhomocysteinemia, hypomethioninemia and absence of methylmalonicaciduria, which is present in cobalamin metabolism disorders. The disease onsetis usuallyin the neonatal period or childhood with the occurrence of neurological symptoms such as encephalopathy, psychomotor delay, gait disorder and epilepsy, all of which may also be associated with thrombotic events [2]. Neonatal formsare usually more severe [1] and are related to the lowest level of residual MTHFR activity [3, 4]. Patients with adolescent/adult onset are rare, with a very heterogeneous neurological presentation. They suffer from spastic paraparesis, psychotic episodes, cognitive disorder, and relapsing encephalopathy [5]. Metabolic therapeutic strategies aim at (i) enhancing methionine synthesis (using B9 and B12 vitamins), (ii) bypassing methionine synthase using betaine (cofactor of another enzyme involved in homocysteine remethylation), and (iii) supplementing methionine if needed [6, 7]. Metabolic therapies were shown to be effective in children and adults to stop disease progression, and sometimes improve neurological disabilities [7]. Few cases of adolescent/adult onset of MTHFR deficiencies are accompanied by epilepsy, and those are usually associated with additional neurological symptoms [5]. Here we report a 32 years-old male patient with pharmaco-resistant frontal epilepsy as the unique manifestation of MTHFR deficiency for the past 14 years, and for whom metabolic treatment initiated 6 years after his first seizure allowed strong reduction of anti-epileptic drugs without recurrence of any seizure. His sister, who also suffered initially from isolated epilepsy, was only started on metabolic treatment 14 years after her initial seizure, and hence developed additional clinical features of MTHFR deficiency. The literature on adolescent/adult onset MTHFR deficiency was also reviewed, with a focus on epilepsy, for characterization ofthe clinical presentation, evolution and response to metabolic treatment.

## Case reports

**Fig. 1:**
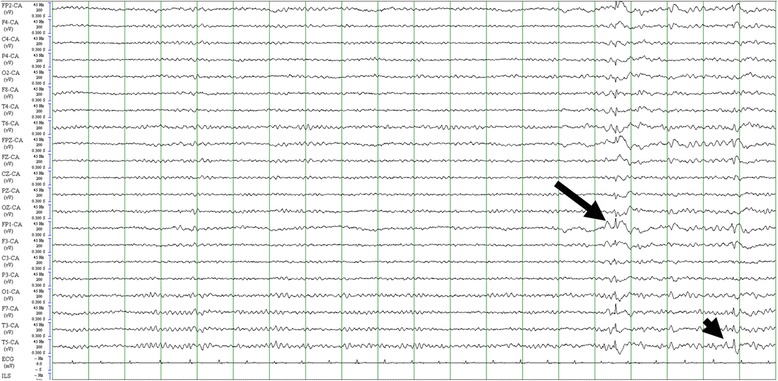
Electroencephalogram (patient n°1). Bilateralfronto-temporal spike-and-waves (arrow), followed by a left temporo-frontal spike (arrow head) on a symmetric alpha (8 Hz) background activity (longitudinal montage)

Patient n°1 (32 year-old man) born from non-consanguineous parents had a normal psychomotor development during childhood but with low scholastic performances and an attention deficit from the age of 14. At 18 years-old, the patient experienced his first epileptic seizure, described as an abrupt arrest of speech, with stereotypical motor behaviors followed by convulsive seizures. Several identical seizures occurred, requiring three antiepileptic drugs to decrease their frequency to two per year: lamotrigine (200 mg/day), valproic acid (1000 mg/day), and clozabam (10 mg/day). Neurological examinationwas normal except for mild cognitive impairment (Mini Mental State Examination -MMSE- 25/30, Frontal Assessment Battery -FAB- 16/18), and subtle upper motor neuron signs in lower limbs. The EEG recorded the presence of left fronto-temporal spike-and-waves on a bilateral and symmetric alpha (8 Hz) background activity (Fig. 1). The brain MRI was normal. At 24 years of age, homocysteinemia, tested because found highly increased in his sister, was found abnormally high at 193 μmol/l (4.5 < *N* < 13.5 μmol/l), whereas plasmatic folate levels were normal with no macrocytic anemia. Plasmatic methionine was low (20 < *N* < 30 μmol/l). The EMG, ophthalmological examination, and echocardiography, performed to investigate disorders associated to hyperhomocysteinemia from genetic causes, were all normal (not shown). Genetic analysis of the *MTHFR* gene identified two heterozygous point mutations: (i) amissense mutation in exon 7 (c.1162C > T, p.(Arg388Cys)); (ii) a stop-loss mutation in exon 12 (c.1970G > C, p.(*657Serext*50)). A heterozygous c.665C > T, p.(Ala222Val) polymorphism in exon 5 corresponding to the c.677C > T nucleotide change according to [8] was also found. The missense mutation in exon 7 affected a highly conserved residue, and was predicted to be deleterious by in silico analyses with SIFT, PolyPhen and Mutation taster. This mutation has never been associated to MTHFR deficiency previously. The stop-loss mutation, previously reported [9], was predicted to lead to the addition of 50 residues at the end of the protein. Genetic analysis showed that the father carried the missense in exon 7 whereas the mother carried the stop-loss in exon 12 (and the c.665C > T polymorphism in exon 5), thus confirming the diagnosis of MTHFR deficiency with compound heterozygosity. Metabolic treatment started at the age of 24, with folinic acid (50 mg per day), betaine (12 g per day), and cyanocobalamin (2 mg per week). This allowed a sustained 50% reduction in homocysteinemia to 80 μmol/l.Valproic acid and clozabam were progressively stopped and lamotrigine dose was decreased to 100 mg a day. The patient has been followed for 5 years after valproic acid stop and for 18 months since the decrease of lamotrigine to 100 mg, and has shown no recurrence of seizure. Since metabolic treatment was introduced, he reported clear improvement in cognition, mainly memory and attention, and was able to live independently from his parents and work as a gardener.

**Fig. 2:**
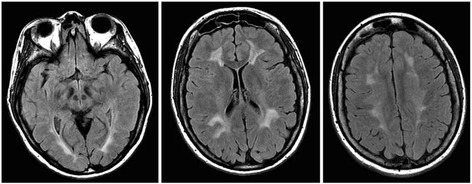
Brain MRI showing white matter changes (patient n°2). Periventricular and subcortical hyper signals with sparing of the U fibers (Axial T2 FLAIR)

His sister (patient n°2, 40 year-old) had normal school performances during adolescence despite behavioral disturbances such as obsessions and addiction to cannabis. Similarly to her brother she presentedcomplex partial seizures at 18 years old (same ictal semiology) but never with generalization. Under valproic acid treatment, she had no recurrence of seizures. At 27 years, she experienced sub-acute neurological symptoms with drowsiness, cognitive disorder, visual hallucinations and paraparesis from central origin, with spontaneous partial improvement. She was again hospitalized at 32 for sub-acute worsening of the same symptoms. MMSE was measured at 22/30. Brain MRI showed diffuse periventricular and subcortical hypersignals of the white matter (Fig. 2). Spinal cord MRI showed a bilateral posterior-lateral hyper-signal at C2 level. EEG showed a slow background theta activity and generalized paroxysmal discharges of spike-and-waves. To investigate an unexplained tachycardia, a contrasted CT thoracic angiography was performed and showed a bilateral pulmonary embolism. Homocysteinemia, tested because of association of thrombosis and unexplained encephalopathy, was found elevated at 130 μmol/l, with decreased plasmatic methionine level, and decreased plasmatic folate levels (2.1 nmol/l; 6 < *N* < 40). Electromyography (EMG) showed a pure motor axonal neuropathy. Echocardiography of the supra aortic trunks was normal. The genetic analysis confirmed that she carried the *MTHFR* mutations detected on her brother. At 32 years old, a metabolic treatment was initiated with folinic acid (75 mg per day), betaine (12 g per day), and cyanocobalamin (1 mg per day), which allowed to keephomocysteinemia stable at levels around 70 μmol/l and improved her clinical condition (gait, cognition, behavior). Valproic acid was then progressively discontinued in few months. At 40 years of age, the patient has had no recurrence of seizures. Unlike her brother she still has gait difficulties with paraparesis and cannot walk without support. After 4 years of metabolic treatment a cerebral MRI did not show worsening signs (not shown).

## Review

A literature review was performed on adolescent/adult onset MTHFR deficiencies. Patients assessed were those identified by Froese et al. [1] who undertook a thorough compilation of all MTHFR deficient patients reported in the literature (*N* = 192 patients from 171 families) [1]. The selection criteria were the following: (i) patients with genetic confirmation of MTHFR deficiency; and (ii) onset of neurological symptoms occurring after 10 years of age (however patients who presented mild learning disabilities before the age of 10 were also included, as many of those patients did not have a specific neurologic evaluation during childhood). Asymptomatic patients were not included but were notified if they were siblings of a reported patient in the table compiling main demographic and clinical characteristics of reviewed patients [see Additional file 1 - legend]. Among 192 patients assessed by Froese et al. [1], 163 had MTHFR mutations, and 22 of them were eligible for this review. Patients were excluded when their age of neurological onset was not known (*n* = 19) or when it occurred before the age of 10 (*n* = 122). Clinical, biochemical, and radiological characteristics of the 22 patients and of the two patients reported here are described in Table 1 (combined data). Additional tables compile the individual data of all 24 patients included in this review [see Additional file 1], as well as their mutations [see Additional file 2], respectively.

**Table 1:** Characteristics of adolescence/adult onset MTHFR deficient patients (*N* = 24 patients)

Mean age at neurological onset (*n* = 24)	22.4 (+/− 12.1, 11–54)	
Age at diagnosis (*n* = 21)	28.8 (+/− 15.3, 11–67)	
Age at description (*n* = 24)	30 (+/− 16.2, 12–69)	
Thrombosis	5/24 (21%)	
Mild learning disabilities	6/21 (29%)	
Acuteness of occurrence of at least one neurological symptom^a^	11/20 (55%)	
Neurological presentation^b^	Gait disorder 11/24 (46%)	LL Weakness 10/10 (100%). 3/10 associated with UL weakness. 1/10 with left sided weakness
		UMN signs 7/7 (100%)
		Spasticity 5/6 (83%)
		Peripheral Neuropathy 3/4 (75%)
		Ataxia 4/8 (50%)
	Epilepsy 7/24 (29%)	GTCS 4/7 (57%), 1 with myoclonus mimicking JME
		Absence 1/7 (14%)
		Focal seizures 2/7 (28%)
	Cognitive decline 5/24 (21%)	
	Psychosis 3/24 (12%)	
	Encephalopathy 1/24 (4%)	
	Stroke 1/24 (4%)	
Neurological symptoms till last follow up	Gait disorder 23/24 (96%)	LL Weakness 21/23 (91%). 6/23 associated with UL weakness. 1/10 with left sided weakness
		UMN signs 19/19 (100%)
		Spasticity 14/17 (82%)
		Peripheral Neuropathy 10/14 (71%)
		Ataxia 7/20 (35%)
	Epilepsy 12/24 (50%)	GTCS 5/9 (55%), 1 with myoclonus in a context of PME (same patient presenting as JME)
		Absence 1/9 (11%)
		Focal seizures 3/9 (33%)
	Cognitive decline 17/23 (74%)	
	Psychosis 4/24 (17%)	
	Encephalopathy 6/20 (30%)	
	Other	Obsessions (*n* = 1), dysarthria (*n* = 1), myoclonus (*n* = 1, with PME), paresthesia (*n* = 1), episodic diplopia (*n* = 1), reduced visual acuity (*n* = 1), urinary and fecal incontinence (*n* = 1), anorexia with progressive withdrawal (*n* = 1), tremor (*n* = 1), UL dysmetria (*n* = 1), coma (*n* = 1).
Delay between first neurological manifestation and occurrence of other neurological symptom^c^	2.8 +/− 2.9, 0–9	
Evolution under treatment	Improvement 15/18 (83%)	
	Stabilization 3/18 (17%)	
Cerebral MRI	White matter abnormalities 12/17 (70%)	
	Normal 3/17 (18%)	
	Cerebral atrophy 7/17 (41%)	
Spinal cord MRI	Normal 3/6 (50%)	
	SCA 2/6 (33%)	
	PCHS 1/6 (17%)	
Biological data	Initial homocysteinemia (*n* = 16)	177.3 +/− 49.5, 115–320
	Initial methioninemia	Low 13/17 (77%), Normal 4/17 (23%)
	Homocysteinemia after treatment (*n* = 13)	76.1 +/−22.2, 50–118

Encephalopathy was defined as an acute or sub-acute onset of cognitive decline with drowsiness and/or confusion

^a^Seizures and strokes were not considered in this row

^b^Four patients had mixed neurological presentations and were counted twice (patientsn°9; 15; 16; and 19)

^c^Symptoms considered: gait disorder, cognitive disorder, epilepsy, psychosis, encephalopathy, and stroke

Among the 24 patients with adolescent/adult onset MTHFR deficiency, 12 patients suffered from epilepsy (50%), with different epileptic syndromes: generalized tonic clonic seizures (*n* = 4), focal seizures (*n* = 3), absence seizures (n = 1), juvenile myoclonic epilepsy (JME) then progressive myoclonic epilepsy (PME) (n = 1), and not documented in other patients (n = 3). Epilepsy was the first manifestation of disease in 7/12 patients (58%), isolated in 6 patients. Sufficient clinical and EEG data to correctly describe the epileptic syndrome were only available for three patients (5, 6 and 10). Patient n°5, a 15 year-old girl, experienced absence type seizures at 13.5 years of age which were successfully treated by sodium valproate. Six months after epilepsy onset, she developed a spastic paraparesis and cognitive difficulties, associated with white matter hypersignals on MRI. Folic acid at 25 mg/day stopped the seizures and reduced MRI white matter abnormalities. Patient n°6, a 17 year-old girl, suffered from mild learning disabilities and had her first seizures characterized by morning myoclonic jerks at 14 years of age. EEG revealed a 4-Hz polyspike waves consistent withJME. Although sodium valproate was efficient during several months, levetiracetam treatment had to be added in order to control her seizures. By the age of 15 she presented paraparesis and ataxia, cognitive decline, and increased seizures frequency with slowing and disorganization of the EEG, which prompted a diagnosis of PME. Under betaine 6 g/day, folinic acid 25 mg/day, and methionine 20 mg/kg/day, all symptoms improved. Seizure frequency decreased from daily to every few months, although the patient still required a triple anti-epileptic treatment (zonisamide, lamotrigine, levetiracetam). Patient n°10 presented generalized tonic-clonic seizures at 19 years-old which were successfully treated by phenytoin, without preventing the onset of leg weakness 2 months after his first seizures. EEG demonstrated predominant theta waves without paroxysmal discharges. Brain MRI revealed diffuse bilateral hyperintensities in the deep white matter. The authors suggested that phenytoin may have precipitated MTHFR deficiency symptoms by aggravating the remethylation impairment. Betaine 6 g/day, folic acid 15 mg/day and pyridoxal phosphate 30 mg/day allowed clinical and radiological improvement, and the patient did not experience recurrence of seizures under zonisamide.

**Fig. 3:**
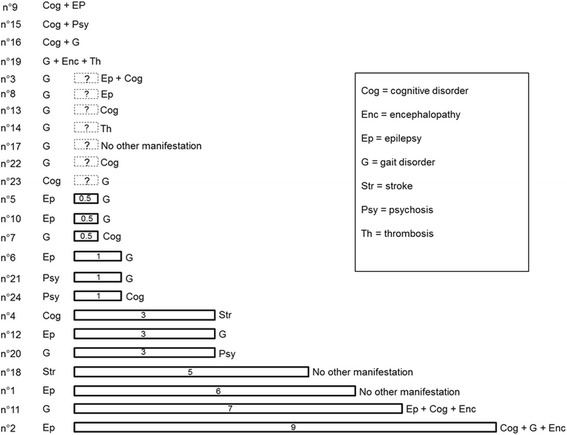
Initial clinical presentation and evolution of symptoms in adolescence/adult onset MTHFR deficiency (*N* = 24 patients). The initial clinical symptom(s) is/are indicated on the left. The delay for onset of other symptoms is represented by the box length, and within the box (in years), followed by the nature of the symptoms. Patients were classified from the shortest to the longest delays of onset of other symptoms

Overall among all 24 reviewed patients, the mean age of onset of neurological symptoms was 22.4 year-old (+/− 12.1, 11–54), excluding mild learning disabilities reported in 29% of patients (6/21) (Table 1). The first neurological manifestations were heterogeneous: gait disorder (11/24, 46%), epilepsy (7/24; 29%), cognitive decline (5/24; 21%), psychosis (3/24; 12%), encephalopathy (1/24; 4%), and stroke (*n* = 1; 4%). A total of 21% of patients (5/24) suffered from thrombosis (venous or arterial). Globally, gait disorder was the most prominent symptom occurring in 96% of patients (23/24), mainly due to lower limbs weakness (21/23; 91%) either from central (19/19; 100% ofpatients had upper motor neuron signs including 14/17–82%-with lower limbs spasticity), or peripheral origin (10/14; 71%had a peripheral neuropathy). Ataxia was less frequent (7/20; 35%). Cognitive decline was also frequently found (17/23; 74%). Four patients had psychotic symptoms (4/24; 17%). Figure 3 shows temporal aspects of onset of different neurological and thrombotic symptoms. Twenty-four percent of patients (4/17) initially had at least two symptoms, whereas 41% (7/17) only suffered from one symptom for at least 3 years. Most patients had periventricular white matter abnormalities (12/17; 71%). Six patients had a spinal cord MRI, one showed bilateral posterior-lateral hypersignal, and two evidenced spinal cord atrophy. Homocysteinemia was strongly increased in all patients (mean value = 177.3 μM +/− 49.5; range: 115–320), whereas methioninemiawas low in 77% of patients (13/17). Among18 metabolically treated patients (no data for the 6 remaining patients), 83% (15/18) improved at least partially while 17% (3/18) remained stable. No patient presented clinical worsening after metabolic treatment was introduced. Mean length of follow up after initiation of metabolic treatment (always at diagnosis) was 3.7 years +/− 4.5 (0–16). Metabolic treatment allowed to halve homocysteinemia (mean homocysteinemia under treatment was 76.1 μM +/− 22.2, 50–118). Patients received B9 vitamins (18/18; 100%), B12 vitamins (16/18; 89%), betaine (15/18; 83%), B6 vitamins (8/18; 44%), methionine (3/18; 17%), riboflavin (2/18; 11%), and thiamine (1/18; 5%), sometimes in a complex temporal sequence. Two patients improved upon B9 supplementation, with or without B12, whereas four patients needed adjunction of betaine to B9/B12 vitamins to further decrease homocysteine levels. Genotype/phenotype correlation was hard to predict, as four adolescent/adult onset patients [see in Additional file 1] had a sibling with a more severe disease beginning in childhood, whereas one patient with neurological onset at 26 years old had a sibling asymptomatic at 37 years old with the same mutations.

## Conclusions

We report the cases of two young adult siblings who experienced epilepsy as the only symptom of MTHFR deficiency during 14 years (including 8 under metabolic treatment) for one and 9 years for the other one. They harbored the stop-loss c.1970G > C mutation that replaces the stop codon by a serine, extending the MTHFR protein by 50 additional amino acids at its C-terminal segment. This stop-loss mutation was previously reported as homozygous in 2 severely affected French patients with early onset disease (< 1 year old) [9]. In our patients the allele with this stop-loss mutation also carried the c.665C > T, p.(Ala222Val) polymorphism in exon 5 (corresponding to the c.677C > T nucleotide change according to [8]), that was previously suggested to worsen MTHFR deficit for patients carrying other *MTHFR* variants [9]. The other mutation we identified (c.1162C > T) has never been associated with MTHR deficiency. Its mutation frequency in control databases is similar to that already reported for other MTHFR causing mutations [see Additional file 2]. Since our two patients first experienced symptoms in adulthood, it is likely that the c.1162C > T mutation does not severely affect MTHFR enzymatic activity, that was shown to be correlated to the severity of the disease [1]. Since the mutation is located outside the catalytic domain of the protein, lying within the predicted S-adenosyl methionine (SAM)-binding site, the protein could display altered binding to SAM while still retaining some residual enzymatic activity [10].

Froese and al. compared genotypes of patients with MTHFR deficiency according to early onset (< 1 year old; *n* = 64) versus late onset (> 1 year old; *n* = 51) [1]. They classified genotypes in seven categories according to the type of mutations (missense/splicing/other) and location of mutations (catalytic domain versus regulatory domain). They found a significant difference only for 2/7 genotype categories: late onset patients had more frequently two missense mutations located in the regulatory domain (14% vs 3%), and less frequently two splicing mutations in the regulatory domain (4% vs 19%). Missense mutations only correlate with a milder phenotype when both located in the regulatory domain, as two missense mutations in the catalytic domain were found equally in early-onset (28%) and late-onset (29%) patients. For the sub-category of very late onset patients (> 10 years old; *n* = 24) reviewed here, the data fit with those findings: 29% of patients had two missense mutations in the catalytic domain, 17% two missense mutations located in the regulatory domain, and 0% two splice mutations in the regulatory domain. Concerning clinical variability among siblings, it is interesting to observe that patient n°7, who suffered from a gait disorder since 15 years old, had an asymptomatic 37 year-old brother harbouring the same mutations. On the other hand, four other patients we included in our manuscript (n°5, 15, 19, 20) had siblings who had an earlier pediatric onset, however never < 1 year old. Therefore, the same genotype can lead to some limited variability of the clinical expression of the disease.

The review of all 24 patients shows that epilepsy occurs in 50% of adolescent/adult onset MTHFR deficient patients with a highly variable phenotype and a variable response to anti-epileptic drugs. The core symptom was gait disorder (96%) from both central and peripheral etiologies. Mode of onset was also variable, some patients experiencing sub-acute onset of symptoms, sometimes following chronic evolution of symptoms. Thrombotic events were not as frequent (5/24; 21% of patients) as reported in homocystinuriadue to cystathionine beta synthase (CBS) deficiency [11]. Even though almost all patients (21/24) ended up suffering from a combination of neurologic symptoms, at the onset, 76% of them (13/17) suffered from a single symptom. The delay from onset to occurrence of a second symptom could be as long as 9 years (patient n°2). The delay from onset to diagnosis was rather long (mean 5.75 years). Only two patients were diagnosed when only suffering from a single symptom, including our patient n°1 who was tested for homocysteinemia after the diagnosis of his sister. Brain MRI can help to achieve diagnosis, but thewhite matter changes observed arenot a constant or a specific sign.

Metabolic treatment is mainly based on B9, B12 vitamins, and betaine. Almost all patients have received those 3 components simultaneously (15/18). All five epileptic patients with data regarding evolution under metabolic treatment had decreased frequency and intensity of seizures, which allowed a decrease or a discontinuation of antiepileptic treatment. Among our case reports, patient n°1, had his epilepsy resolved and remained free of other symptoms for 8 years, until last follow-up. Interestingly, his older sister (patient n°2), diagnosed at a later age, experienced gait difficulties 9 years after the appearance of epilepsy, suggesting that the early start of metabolic treatment of her brother prevented the worsening of his disease. Among 18 patients for whom evolution under metabolic treatment was reported, all stabilized or improved clinically, while their homocysteinemia levels, although strongly reduced, never completely normalized. However, very few had a complete disappearance of their symptoms due to irreversible neurological damage accumulating over time, highlighting the need for shorter diagnostic delays in MTHFR deficiency.

This could be achieved if homocysteinemia was tested earlier as a screening test for MTHFR deficiency. Values reported in adolescent/adult onset MTHFR deficiency were consistently above 100 μM (4.5 < *N* < 15), even for very late onset patients, strongly evoking a genetically-sustained metabolic defect, and notvitamins or renal filtration deficiencies, which can also be associated withhyperhomocysteinemia.

Homocysteine is likely to promote thrombotic events, but it is not known why such events are far less frequent in MTHFR deficiency than in classic homocystinuria despite both deficiencies being associated with similar homocysteinemia levels [11]. Hypomethioninemia may decrease global methylation reactions in the central nervous system, hence possibly affecting myelin, as attested by white matter abnormalities often found in cerebral MRIs of MTHFR deficient patients [12].

In conclusion, these two patients broaden the phenotypic spectrum of epilepsy in adolescent/adult onset MTHFR deficiency. The literature review showed that epilepsy, and other isolated neurological symptoms like spastic paraparesis or cognitive decline, may be the unique manifestations of MTHFR deficiency during several years. Even though adolescent/adult onset MTHFR deficiency is a rare disease, it is a treatable one, for which metabolic treatment comprising B9, B12 and betaine can prevent disease progression and promote improvement. Assessment of homocysteinemiashould be performed in selected patients even if suspicion of MTHFR deficiency is low. We suggest that plasma homocysteine levels be tested in case of occurrence of the following symptoms with unknown etiologies:unexplained epilepsy with or without normal brain MRI, spastic paraparesis, motor predominant peripheral nerve disease with central signs, young onset cognitive disorder, encephalopathy, atypical psychosis (with visual hallucinations, cognitive disorder, drowsiness), and young onset thrombosis. In case of hyperhomocysteinemia, metabolic treatment should be started without delay.

## Additional files

Additional file 1: ‘*Clinical, biochemical, and radiological characteristics of 24 adolescent/adult onset MTHFR deficient patients*’. This large table compiles the main demographic and clinical characteristics of patients with adolescent/adult onset MTHFRdeficiency. (DOCX 79 kb)

Additional file 2: ‘MTHFR mutations of 24 adolescent/adult onset MTHFR deficiency patients from the literature [1] and presently reported’. This table compiles the mutations of all 24 MTHFR deficient patients with an adolescent/adult onset that were reviewed in this manuscript. (DOCX 23 kb)

## Abbreviations

5MTHF: 5- methyl-tetrahydrofolate
B12: Vitamin B12, ie, cobalamin
B9: Vitamin B9, ie, folic acid
C2: 2nd cervical vertebrae
EEG: Electroencephalography
EMG: Electromyography
FAB: Frontal assessment battery
GTCS: Generalized tonico clonic seizure
JME: Juvenile myoclonic epilepsy
LL: Lower limbs
MMSE: Mini mental state examination
MRI: Magnetic resonance imagery
MTHFR: 5,10-Methylene-tetrahydrofolate reductase
PME: Progressive myoclonic epilepsy
UL: Upper limbs
UMN: Upper motor neuron
