# Supplementary material for: Adolescence/adult onset MTHFR deficiency may manifest as isolated and treatable distinct neuro-psychiatric syndromes
Source: Orphanet J Rare Dis. 2018 Feb 1;13:29. doi: 10.1186/s13023-018-0767-9 (PMC5796584; doi:10.1186/s13023-018-0767-9)
Supplement: Supplementary file 1 — ‘Clinical, biochemical, and radiological characteristics of 24 adolescent/adult onset MTHFR deficient patients’. This large table compiles the main demographic and clinical characteristics of patients with adolescent/adult onset MTHFRdeficiency. (DOCX 79 kb) [file 13023_2018_767_MOESM1_ESM.docx]

| N° | Demography | | | | Symptomatology | | Neurology | | | | | | | Psychiatry/Cognition | | | | Vascular/others | | Imaging/biochemistry | | | Evolution under metabolic treatment | Ref |
| --- | --- | --- | --- | --- | --- | --- | --- | --- | --- | --- | --- | --- | --- | --- | --- | --- | --- | --- | --- | --- | --- | --- | --- | --- |
|  | Sex | Age at onset | Age at diagnosis | Age at description | Initial symptom(s) | Acuteness | Epilepsy (age) | Gait disorder (age) | Spasticity/UMN signs | Tendon reflexes | Weakness | Ataxia | Peripheral neuropathy | Psychosis (age) | Cognitive decline (age) | Encephalopathy | Mild learning disabilities | Thrombosis | Other symptoms | Cerebral and SC MRI | Homocysteine initial/after treatment (µM) | Methioninemia |  |  |
| 1 | M | 18 | 24 | 32 | Focal seizures | - | + (18) | - | -/+ | N | - | - | - | - | - | - | + | - | No | Normal (C) | 193/80 | N | Impr. | This study |
| 2 | F | 18 | 32 | 40 | Focal seizures | + | + (18) | + (27) | +/+ | Brisk (LL) | + (LL) | + | + | - | + (27) | + (27) | - | + (32) DVT, PE | Behavior disorder (obsession,..) | PVLeu; SC : PCHS | 129/70 | 7 µM (low) | Impr. | This study |
| 3 | M | 11 | 12 | 12 | Gait disorder | - | +  Focal | + (11) | ND/+ | ND | + | + | + | - | + | ND | - | - | No | ND | ND | ND | Impr. | [1, 2] |
| 4 | F | 12 | ND | 16 | Cognitive disorder | ND | + (16) | + (16) | +/+ | ND | +  (LL) | ND | ND | - | + (12) | ND | ND | + (15) Stroke | No | ND | ND | ND | ND | [3] |
| 5 (1) | F | 13.5 | 15 | 27 | Absence seizures | - | + (13.5) | + (14) | +/+ | ND | +  (UL/LL) | - | ND | - | - | - | + | - | Dysarthria | PVLeu | ND | ND | Impr. | [4] |
| 6 | F | 14 | 15 | 17 | GTC seizures | - | + (14) | + (15) | +/+ | ND | +  (LL) | + | - | - | + | - | + | - | Myoclonus (PME) | CA; SC:N | 152.14/80.69 | 6.2 µM (low) | Impr. | [5] |
| 7 (2) | M | 15 | 26 | 27 | Gait disorder | - | + (26)  GTC seizures | + (15) | +/+ | N | +  (UL/LL) | - | ND | - | + (15) | - | - | - | Paresthesiae, episodic diplopia, reduced VA | ND | ?/minus50% | Low | Impr. | [6] |
| 8 | M | 16 | ND | 16 | Gait disorder | ND | + | + (16) | ND | ND | + | ND | ND | - | - | ND | ND | - | No | ND | ND | ND | ND | [1, 7, 8] |
| 9 | F | 17 | 35 | 37 | Cognitive disorder, GTC seizure | + | + (17) | + (35) | ND/+ | brisk | - | + | - | - | + (17) | - | - | - | No | Cleu (posterior) | 215/118 | 7.9 µM (low) | Impr. | [9] |
| 10 | M | 19 | 20 | 21 | GTC seizures | + | + (19) | + (19) | +/+ | weak | +  (UL/LL) | - | + (reduced velocity in LL) | - | + (20) | + (20) | - | - |  | CLeu | 219.7/100.8 | 7.3 µM (low) | Impr. | [10] |
| 11 | ND | 20  (3) | 27 | 30 | Gait disorder | + | + (27) | + (20) | +/+ | ND | +  (LL) | - | - | - | + (27) | + (27) | - | - | urinary and faecal incontinence | CA | 155/94.9 | 18 µM (N) | ND | [11] |
| 12 | F | 37 | 53 | 69 | GTC seizures | - | + (37) | + (40) | +/+ | weak (ankle) | +  (LL) | - | + SMP | - | + | - | - | - | No | PVLeu (posterior), CA, SCA | 140/56 | 0.16 mg/dL (low) | Impr. | [12] |
| 13 | M | 11 | 11 | 15 | Gait disorder | + | - | + (11) | +/+ | ND | +  (UL/LL) | + | + | - | + | - | + | - | No | CLeu | 186 | 6 (low) | Stab. | [13] |
| 14 | F | 14 | ND | 14 | Gait disorder | ND | - | + (14) | ND | ND | +  (LL) | + | ND | - | - | - | + | + DVT, PE | No | ND | ND | ND | ND | [1] |
| 15 (4) | F | 12 | 15 | 20 | Cognitive disorder, Psychosis (hallucinations) | + | - | + (15) | -/+ | absent | +  (UL/LL) | - | + | + (12) | + (12) | + (15) | - | - | Anorexia, tremor, progressive withdrawal | ND | ND | ND | Impr. | [1, 14] |
| 16 | ND | 16 | 16 | 16 | Gait and cognitive disorders | - | - | + (16) | ND | ND | ND | ND | ND | - | + (16) | ND | ND | - | No | CLeu | ND | ND | ND | [15] |
| 17 | M | 18 | 18 | 18 | Gait disorder | + | - | + (18) | ND | ND | + | ND | ND | - | - | - | - | - | No | ND | 186/107 | 3.8 µM (low) | ND | [16] |
| 18 | M | 20 | 25 | 27 | Stroke (veinous) | + | - | + (20) | ND | ND | + (left sided) | - | ND | - | - | - | - | + (20) | No | Infarct | 115/50 | 11 µM (N) | Stab. | [17] |
| 19 (5) | F | 27 | 27 | 28 | Gait disorder, encephalopathy | + | - | + (27) | -/+ | weak | + | - | ND | - | + | + (27) | + | + (27) | Dysmetria UL | N (brain and SC) | 320 | 3 µM (low) | Impr. | [18] |
| 20 (6) | M | 28 | 31 | 32 | Gait disorder | + | - | + (28) | +/+ | ND | + (UL/LL) | + | ND | + (31) | ND | - | - | - | No | PVLeu, CA | 186 | 14 µM (N) | Impr. | [19] |
| 21 | F | 29 | 34 | 35 | Psychosis | - | - | + (30) | +/+ | weak (ankle) | + (LL) | - | + SMP | + (29) | + | - | - | - | No | PVLeu (posterior), CA, SCA | 150/60 | O.075 mg/dL (low) | Stab. | [12] |
| 22 | F | 40 | 45 | 46 | Gait disorder | - | - | + (40) | +/+ | weak (ankle) | + (LL) | - | + SMP | - | + | - | - | - | No | PVLeu (posterior), CA | 185/74 | 0.12 mg/dL (low) | Improvement | [12] |
| 23 | M | 50 | 67 | 68 | Cognitive disorder | - | - | + | +/+ | weak (ankle) | + (LL) | - | + SMP | - | + (50) | - | - | - | No | PVLeu (posterior), CA | 152 | 0.09 mg/dL (low) | Improvement | [12] |
| 24 | F | 54 | 56 | 57 | Psychosis (hallucinations) | + | - | + (55) | +/+ | absent | + (LL) | - | + (axonal sensory neuropathy) | + (54) | + (55) | + (56) | - | - | coma | PVLeu; SC:N | 170/85 | 15 µM (low) | Impr. | [20] |

**Additional file 1 - Clinical, biochemical, and radiological characteristics of 24 adolescent/adult onset MTHFR deficient patients.**

A total of 22 patients from the literature [21] and 2 patients presently reported were included.

Encephalopathy was defined as an acute or subacute onset of cognitive decline with drowsiness and/or confusion.

(1) This patient has a brother with MTHFR deficiency with mental retardation in whom gait disorder began at 14 years old

(2) This patient has a brother with MTHFR deficiency, asymptomatic at 37 years old

(3) "Young adult", supposedly around 20 years old

(4) This patient has a sister with MTHFR deficiency with mental retardation

(5) This patient has a sister with MTHFR deficiency, age of onset at 5 years old (mental retardation, gait disorder, epilepsy)

(6) This patient has a brother with MTHFR deficiency and mental retardation

PVLeu: periventricular leucopathy; CLeu: Cerebral Leucopathy; Impr.: improvement; GTC: Generalized Tonic Clonic; PME: Progressive Myoclonic Epilepsy; CA: Cerebral Atrophy; SC: Spinal Cord; SCA: Spinal Cord Atrophy; N: Normal; ND: Not Documented; DVT: Deep Vein Thrombosis; PE: Pulmonary Embolism; UL: Upper Limbs; Stab: Stability; SMP: Sensory Motor Polyneuropathy; LL: Lower Limbs; UL: Upper Limbs; UMN: Upper Motor Neuron

**REFERENCES**

1. Goyette P, Frosst P, Rosenblatt DS, Rozen R. Seven novel mutations in the methylenetetrahydrofolate reductase gene and genotype/phenotype correlations in severe methylenetetrahydrofolate reductase deficiency. American journal of human genetics. 1995;56(5):1052-9.

2. Boss GR, Erbe RW. Decreased rates of methionine synthesis by methylene tetrahydrofolate reductase-deficient fibroblasts and lymphoblasts. The Journal of clinical investigation. 1981;67(6):1659-64.

3. Sibani S, Leclerc D, Weisberg IS, O'Ferrall E, Watkins D, Artigas C, et al. Characterization of mutations in severe methylenetetrahydrofolate reductase deficiency reveals an FAD-responsive mutation. Human mutation. 2003;21(5):509-20.

4. Tallur KK, Johnson DA, Kirk JM, Sandercock PA, Minns RA. Folate-induced reversal of leukoencephalopathy and intellectual decline in methylene-tetrahydrofolate reductase deficiency: variable response in siblings. Developmental medicine and child neurology. 2005;47(1):53-6.

5. D'Aco KE, Bearden D, Watkins D, Hyland K, Rosenblatt DS, Ficicioglu C. Severe 5,10-methylenetetrahydrofolate reductase deficiency and two MTHFR variants in an adolescent with progressive myoclonic epilepsy. Pediatric neurology. 2014;51(2):266-70.

6. Haworth JC, Dilling LA, Surtees RA, Seargeant LE, Lue-Shing H, Cooper BA, et al. Symptomatic and asymptomatic methylenetetrahydrofolate reductase deficiency in two adult brothers. American journal of medical genetics. 1993;45(5):572-6.

7. Mudd SH, Uhlendorf BW, Freeman JM, Finkelstein JD, Shih VE. Homocystinuria associated with decreased methylenetetrahydrofolate reductase activity. Biochemical and biophysical research communications. 1972;46(2):905-12.

8. Mudd SH. Biochemical and Biophysical Research Communications. Biochemical and biophysical research communications. 1972;374:559-64.

9. Tamura A, Sasaki R, Kagawa K, Nakatani K, Osaka H, Tomimoto H. [Posterior-predominant leukoencephalopathy which was caused by methylenetetrahydrofolate reductase deficiency and successfully treated with folic acid]. Rinsho shinkeigaku = Clinical neurology. 2014;54(3):200-6.

10. Arai M, Osaka H. Acute leukoencephalopathy possibly induced by phenytoin intoxication in an adult patient with methylenetetrahydrofolate reductase deficiency. Epilepsia. 2011;52(7):e58-61.

11. Bathgate D, Yu-Wai-Man P, Webb B, Taylor RW, Fowler B, Chinnery PF. Recessive spastic paraparesis associated with complex I deficiency due to MTHFR mutations. Journal of neurology, neurosurgery, and psychiatry. 2012;83(1):115.

12. Lossos A, Teltsh O, Milman T, Meiner V, Rozen R, Leclerc D, et al. Severe methylenetetrahydrofolate reductase deficiency: clinical clues to a potentially treatable cause of adult-onset hereditary spastic paraplegia. JAMA neurology. 2014;71(7):901-4.

13. Schiff M, Benoist JF, Tilea B, Royer N, Giraudier S, Ogier de Baulny H. Isolated remethylation disorders: do our treatments benefit patients? Journal of inherited metabolic disease. 2011;34(1):137-45.

14. Freeman JM, Finkelstein JD, Mudd SH. Folate-responsive homocystinuria and "schizophrenia". A defect in methylation due to deficient 5,10-methylenetetrahydrofolate reductase activity. The New England journal of medicine. 1975;292(10):491-6.

15. Sibani S, Christensen B, O'Ferrall E, Saadi I, Hiou-Tim F, Rosenblatt DS, et al. Characterization of six novel mutations in the methylenetetrahydrofolate reductase (MTHFR) gene in patients with homocystinuria. Human mutation. 2000;15(3):280-7.

16. Rummel T, Suormala T, Haberle J, Koch HG, Berning C, Perrett D, et al. Intermediate hyperhomocysteinaemia and compound heterozygosity for the common variant c.677C>T and a MTHFR gene mutation. Journal of inherited metabolic disease. 2007;30(3):401.

17. Kim SJ, Lee BH, Kim YM, Kim GH, Yoo HW. Congenital MTHFR deficiency causing early-onset cerebral stroke in a case homozygous for MTHFR thermolabile variant. Metabolic brain disease. 2013;28(3):519-22.

18. Tonetti C, Ruivard M, Rieu V, Zittoun J, Giraudier S. Severe methylenetetrahydrofolate reductase deficiency revealed by a pulmonary embolism in a young adult. British journal of haematology. 2002;119(2):397-9.

19. Birnbaum T, Blom HJ, Prokisch H, Hartig M, Klopstock T. Methylenetetrahydrofolate reductase deficiency (homocystinuria type II) as a rare cause of rapidly progressive tetraspasticity and psychosis in a previously healthy adult. Journal of neurology. 2008;255(11):1845-6.

20. Michot JM, Sedel F, Giraudier S, Smiejan JM, Papo T. Psychosis, paraplegia and coma revealing methylenetetrahydrofolate reductase deficiency in a 56-year-old woman. Journal of neurology, neurosurgery, and psychiatry. 2008;79(8):963-4.

21. Froese DS, Huemer M, Suormala T, Burda P, Coelho D, Gueant JL, et al. Mutation Update and Review of Severe Methylenetetrahydrofolate Reductase Deficiency. Human mutation. 2016;37(5):427-38.
