# Supplementary material for: Adolescence/adult onset MTHFR deficiency may manifest as isolated and treatable distinct neuro-psychiatric syndromes
Source: Orphanet J Rare Dis. 2018 Feb 1;13:29. doi: 10.1186/s13023-018-0767-9 (PMC5796584; doi:10.1186/s13023-018-0767-9)
Supplement: Supplementary file 2 — ‘MTHFR mutations of 24 adolescent/adult onset MTHFR deficiency patients from the literature [1] and presently reported’. This table compiles the mutations of all 24 MTHFR deficient patients with an adolescent/adult onset that were reviewed in this manuscript. (DOCX 23 kb) [file 13023_2018_767_MOESM2_ESM.docx]

| Nucleotide change (Goyette et al., 1998) | Nucleotide change (HGVS) | Predicted amino acid change | rs numbers (dbSNP) | ExAC browser frequency | Patient Number |
| --- | --- | --- | --- | --- | --- |
| c.148C>T / c.167G>A | c.136C>T / c.155G>A | p.Arg46Trp / p.Arg52Gln | rs138189536 / rs754980119 | 0.0042% / 0.023% | 5 |
| c.167G>A / c.1015C>T | c.155G>A / c.1003C>T | p.Arg52Gln / p.Arg335Cys | rs754980119 / rs748289202 | 0.000076% / 0.000015% | 3 |
| c.167G>A / c.793-1G>A | c.155G>A / c.781-1G>A | p.Arg52Gln / ND | rs754980119 / none | 0.000076% / ND | 6 |
| c.167G>A / c.1081C>T | c.155G>A / c.1069C>T | p.Arg52Gln / p.Arg357Cys | rs754980119 / rs779993607 | 0.000076% / 0.00083% | 14 |
| c.[167G>A; 1178+1G>A] / c.1331C>T | c.[155G>A; 1166+1G>A] / c.1319C>T | [p.Arg52Gln; ND] / p.Ser440Leu | [rs754980119; none] / rs754554624 | [0.000076%; 0%] / 0.0025% | 19 |
| c.358G>A / c.1134C>G | c.346G>A / c.1122C>G | p.Ala116Thr / p.Tyr374* | none / none | 0% / 0% | 10 |
| c.358G>A / c.1134C>G | c.346G>A / c.1122C>G | p.Ala116Thr / p.Tyr374* | none / none | 0% / 0% | 16 |
| c.471C>G / c.1274G>A | c.459C>G / c.1262G>A | p.Ile153Met / p.Trp421* | rs767890671 / none | 0% / 0% | 4 |
| c.482G>A / c.1711C>T | c.470G>A / c.1699C>T | p.Arg157Gln / p.Arg567* | rs121434295 / rs140277700 | 0.0033% / 0.0025% | 7 |
| c.[482G>A; 1551dupA] / c.1551dupA | c.[470G>A; 1539dupA] / c.1539dupA | [p.Arg157Gln; p.Glu514Argfs*24] / p.Glu514Argfs*24 | [rs121434295; none] / none | [0.0033%; 0%] / 0% | 24 |
| c.596C>T / c.596C>T | c.584C>T / c.584C>T | p.Ala195Val / p.Ala195Val | rs760161369 / rs760161369 | 0.0033% / 0.0033% | 11 |
| c.616C>A / c.616C>A | c.604C>A / c.604C>A | p.Pro202Thr / p.Pro202Thr | none / none | 0% / 0% | 13 |
| c.685A>C / c.685A>C | c.673A>C / c.673A>C | p.Ile225Leu / p.Ile225Leu | rs200100285 / rs200100285 | 0% / 0% | 9 |
| c.792+1G>A / NI | c.780+1G>A / NI | ND / NI | none / NI | 0% / NI | 15 |
| c.985C>T / c.985C>T | c.973C>T / c.973C>T | p.Arg325Cys / p.Arg325Cys | rs371085894 / rs371085894 | 0.0025% / 0.0025% | 8 |
| c.1045C>T / c.1553_1554delAG | c.1033C>T / c.1541_1542delAG | p.Arg345Cys / p.Glu514Valfs*23 | rs759031330 / rs764338697 | 0.00083% / 0.0017% | 17 |
| c.1082G>A / c.1616G>A | c.1070G>A / c.1604G>A | p.Arg357His / p.Arg535Gln | none / rs773360881 | 0% / 0.0016% | 20 |
| c.1082G>A / c.1181G>A | c.1070G>A / c.1169G>A | p.Arg357His / p.Gly390Asp | none / none | 0% / 0% | 18 |
| c.1142G>A / c.1142G>A | c.1130G>A / c.1130G>A | p.Arg377His / p.Arg377His | rs750323424 / rs750323424 | 0.0025% / 0.0025% | 21 |
| c.1142G>A / c.1142G>A | c.1130G>A / c.1130G>A | p.Arg377His / p.Arg377His | rs750323424 / rs750323424 | 0.0025% / 0.0025% | 22 |
| c.1153T>C^1^ / c.1547A>G | c.1141T>C^1^ / c.1535A>G | p.Trp381Arg / p.Tyr512Cys | none / none | 0% / 0% | 12 |
| c.1153T>C^1^ / c.1547A>G | c.1141T>C^α^ / c.1535A>G | p.Trp381Arg / p.Tyr512Cys | none / none |  | 23 |
| c.1174C>T / c.1984G>C | c.1162C>T / c.1970G>C | p.Arg388Cys / p.*657Serext*50 | rs200138092 / rs749490263 | 0.022% / 0.00084% | 1 |
| c.1174C>T / c.1982G>C | c.1162C>T / c.1970G>C | p.Arg388Cys / p.*657Serext*50 | rs200138092 / rs749490263 | 0.022% / 0.00084% | 2 |

**Additional file 2 -** **MTHFR mutations of 22 published adolescent/adult onset MTHFR deficiency patients identified from the literature [1] in addition to the two patients presently reported.**

ND: not defined. NI: not identified. ^α^: corrected from the original article

1. Froese DS, Huemer M, Suormala T, Burda P, Coelho D, Gueant JL, et al. Mutation Update and Review of Severe Methylenetetrahydrofolate Reductase Deficiency. Human mutation. 2016;37(5):427-38.
